# Notes on Preparations for the Sick

**Published:** 1912-12

**Authors:** 


### Notes on Preparations for the Sick.

**"Tabloid" Hypodermic Ergamine 0·001 gm.**—BURROUGHS, WELLCOME & Co., London.—Ergamine is a recently-isolated active principle of Ergot. Its most important action is as a stimulant of plain muscle, this action being particularly conspicuous in the case of the uterus.

In rodents Ergamine causes a rise of blood pressure, but in carnivora and monkeys its exhibition gives rise to a fall of blood pressure.

The drug is indicated for use when prompt uterine contraction is desired. The dose advised is one milligramme, to be repeated with caution.

---

**"Tabloid" Ophthalmic Physostigmine Salicylate, gr. 1/4000. "Tabloid" Ophthalmic Pilocarpine Nitrate, gr. 1/3000.**—Burroughs, Wellcome & Co., London.—Two new "Tabloid" ophthalmic products have been added to the list. These are Physostigmine Salicylate, gr. 1/4000, and Pilocarpine Nitrate, gr. 1/3000. They are intended for direct application to the eye without previous solution, and are suitable for use by patients themselves in place of eye drops, having the advance over the latter of ensuring accurate doses. One of the physostigmine salicylate products is approximately equivalent to one drop of a solution of gr. $\frac{1}{8}$ to the ounce, and one of the pilocarpine nitrate products to one drop of a gr. $\frac{1}{6}$ to the ounce solution.

---

**"Tabloid" "Epinine" Compound.**—Burroughs, Wellcome & Co., London.—"Tabloid" "Epinine" Compound contains gr. 1/1000 of "Epinine" (the synthetic substance with adrenine-like action), in combination with suitable small doses of Heroin Hydrochloride, Ipecacuanha, Benzoic Acid and Oil of Gaultheria. It is intended to be sucked so as to secure the local application of these drugs to the mucous membranes of the throat. The product is made with a demulcent base, and dissolves slowly in the mouth, thus ensuring prolonged contact of the medicaments with the affected parts. The vaso-constrictor and astringent action of "Epinine" and the sedative, expectorant and antiseptic effects of the other constituents, make the product a very useful one for congested and irritated conditions of the pharynx and larynx. It should prove particularly valuable to singers and public speakers. The taste is agreeable, and the quantity of each ingredient is such as to allow of frequent repetition of the dose.

---

**"Paroleine" Spray Compound.**—Burroughs, Wellcome & Co., London.—This Spray Compound contains suitable proportions of menthol, chlorbutol and "Eucalyptia" in a basis of "Paroleine," the whole forming an excellently designed preparation to be applied, as a spray, to the nose or throat. The combination is a well-tried one, and has been found exceedingly useful for its sedative, analgesic and antiseptic effects in catarrhal conditions of the respiratory tract generally.

**Malt-Glidine (with Lecithin).**—MENLEY & JAMES, London.—The analysis of this food in the dry form gives per cent. :—

| | |
|---|---|
| Protein .. .. .. .. | 37 |
| Malted Carbohydrates .. .. | 34 |
| Natural Fats .. .. .. | 18 |
| Lecithin .. .. .. .. | 6 |
| Phosphates and other Salts .. | 5 |
| | 100 |

A compound such as this should be of value as a nerve tonic in all debilitated conditions of the nervous and digestive systems, such as neurasthenia and wasting diseases generally. It should be a useful addition to the list of Glidine compounds previously noticed by us. It is quite palatable and inexpensive.

---

**Atophan-Schering** ($C_{16}H_{11}NO_2$).—A. & M. ZIMMERMANN, London.—Another anti-uric-acid drug, having the composition 2 Phenylchinolin + 4 Carbonic Acid, is said to increase the elimination of uric acid to two or four times the usual amount. In gout its effect surpasses that of the aceto-salicylic acid, it is more prompt in its action than colchicum, and it has no injurious effect on heart, stomach, or nerves. It is put up in tablet form, 6 to 8 for a daily dose. The tablets are prepared with cacao, but readily disintegrate in water. It is suggested that sodium bicarbonate in large doses may be given simultaneously,

Any new drug with properties so described must be welcome for the numerous cases of gout, neuralgia, and the various forms of muscular and articular rheumatism, which do not always yield to any one preparation. Excellent results are reported from many continental authorities, but it is advised that as the deposition of uric acid is so decided the drug should not be given in cases where stone is present in the urinary passages.

---

**Cystopurin.**—A. WULFING & Co., London.—This preparation is the outcome of long and careful research. Chemically, it is a double salt of Hexamethylene-tetramine and Sodium Acetate. When taken internally the Sodium Acetate is partially or wholly converted in the body into sodium carbonate, the result being that Cystopurin has the specific action of the Hexamethylene-tetramine on the urinary tract considerably fortified. It changes the constitution of the urine, and produces greater diuresis than its separate ingredients, or any other combination of them.

It is readily soluble in water, has a slight saline taste, and has proved itself to be most efficient in the treatment of bacterial conditions of the urine.

Each tablet contains one gramme, and the dose of two grammes thrice daily is adequate.

---

**Milk-Chocolate Peptonised.**—SAVORY & MOORE, London.—This is a dry preparation (for eating) of the well-known Peptonised Cocoa and Milk.

Its nutrient value is double that of the ordinary chocolate: its flavour is excellent, and it should be easy of digestion.

The Cocoa-Milk preparation has had a long run of popularity, and it continues to be one of the most nourishing and useful preparations for the sick. In the chocolate form its area of utility is likely to be extended to those who are not sick.

---

**Codeonal.**—KNOLL & Co., London.—Each tablet contains $2\frac{1}{2}$ grains, which is equivalent to $2\frac{1}{4}$ grains of di-ethylbarbiturate of sodium and a $\frac{1}{4}$ grain of a similar salt of codeine. The combination is a chemical one, the codeine salt forming definite crystals readily soluble in water. The drug is a prompt hypnotic acting well in half-an-hour, and giving a sleep of six or seven hours' duration in cases where the insomnia is not due to severe pain or dyspnœa. It appeared to suit cases of phthisis with severe irritative cough.

---

**Kaloof Compress.**—HUDSON AND RICKETT, Tottenham, N.—A sterilised aseptic hot compress.

It consists of an asbestos envelope, to one side of which is attached a loofah pad, and to the other side a covering of non-conductible and impervious material. Its uses are as various as the hot fluids in which it may be immersed before being applied to the skin or wound, to which it forms an aseptic hot compress if sterilised by immersion in boiling water, or some boiling antiseptic solution. It may be continued and renewed indefinitely as long as required.

A variety of compresses for different purposes may be obtained, and it is suggested that Kaloof Gloves, Boots and Knee Compresses may replace localised hot air and radiant heat baths. The pads can also be utilised as post-operation dressings.

---

**Neutrigen Tonic Food.**—THE MEDICAL ENTERPRISE SOCIETY, Palace Chambers, Westminster, S.W.—Under this name a proteid food has recently been introduced to the medical profession.

The food is composed of casein, lecithin, and sodium glycerophosphates, and, unlike so many preparations on the market, readily mixes with water to form a mucilage.

It should be specially noted that the article in question is not being advertised to the public, but is being introduced to medical men only. This, and its reasonable price, should commend it to those practitioners who are requiring a similar type of tonic food.

